# Extraradicular Infection and Apical Mineralized Biofilm: A Systematic Review of Published Case Reports

**DOI:** 10.3390/jcm14072335

**Published:** 2025-03-28

**Authors:** Alejandro R. Pérez, Jaime Rendón, P. S. Ortolani-Seltenerich, Yetzangel Pérez-Ron, Miguel Cardoso, Rita Noites, Gaizka Loroño, Gaya C. S. Vieira

**Affiliations:** 1 Department of Endodontics, Rey Juan Carlos University, Alcorcón, 28922 Madrid, Spain; 2 Private Practice, Villa Nova de Gaya, 4400-239 Porto, Portugal; dragayacarolina@gmail.com; 3 Surpreendente Research Group, Villa Nova de Gaia, 4400-239 Porto, Portugal; yepilu28@gmail.com; 4 POPCAD Research Group, Laboratory of Immunodetection and Bioanalysis, Faculty of Dentistry, University of Antioquia, Medellín 050026, Colombia; jaime0978@gmail.com; 5 Department of Dental Pathology and Therapeutics, Faculty of Dentistry, UCAM, 30107 Murcia, Spain; psortolani@gmail.com; 6 Centre for Interdisciplinary Research in Health, Faculty of Dental Medicine, Universidade Católica Portuguesa, 3504-505 Viseu, Portugal; miguelbcardoso@gmail.com (M.C.); rnoites@gmail.com (R.N.); 7 Postgraduate Program in Endodontics, Department of Dentistry, Universidad Europea de Madrid, 28670 Madrid, Spain; gaizkaloro@gmail.com

**Keywords:** extraradicular infection, extraradicular biofilm, like-calculus deposit, wet canals, sinus tract

## Abstract

**Background/Objectives**: Bacterial biofilms on root surfaces outside the apical foramen are linked to refractory apical periodontitis, as microorganisms can survive in extraradicular areas and cause persistent infections. This study aimed to precisely evaluate the relationship between extraradicular biofilm and persistent periapical periodontitis through an overview of case reports. **Methods**: A systematic search of PubMed, Web of Science, Scopus, Embase and ScienceDirect databases was conducted up to June 2023. Keywords included “extraradicular infection”, “wet canal”, “wet canals”, “extraradicular mineralized biofilms”, and “calculus-like deposit”. Only case reports meeting the inclusion criteria were analyzed. **Results**: Fifteen cases of extraradicular infection were identified, involving eight women and six men aged between 18 and 60 years. These cases included nine failed treatments confirmed through complementary methods such as histobacteriologic analysis, scanning electron microscopy (SEM), or polymerase chain reaction (PCR). Among these, four patients (six teeth) exhibited calculus-like deposits. **Conclusions:** Extraradicular biofilm is strongly associated with failed endodontic treatments, leading to persistent infections. A structured decision-making approach is essential. Before considering apical surgery, clinicians should prioritize intraradicular infection control through thorough irrigation, antimicrobial medicaments, and adjunctive disinfection techniques. When extraradicular biofilms or mineralized calculus are present, and symptoms persist after optimal intracanal disinfection, apical surgery should be performed.

## 1. Introduction

Apical periodontitis is an inflammatory disease caused by bacteria colonizing the root canal system [1,2]. The lesions are generally free of microorganisms, but bacteria may invade the periapical tissues and establish an extraradicular infection in some specific circumstances [3]. Although this type of infection is rare, extraradicular bacterial biofilms have been observed in approximately 6% of cases of apical periodontitis, including both untreated and previously treated canals [4]. Certain microorganisms can survive outside the root canal and sustain inflammation in the periapical tissues. The presence of bacteria in the periradicular tissues has already been observed, both incorporating the body of the inflammatory lesion, usually forming cohesive colonies [3], and in the form of biofilm, adhered to the external surface of the root [4].

Extraradicular infection can be dependent or independent of the root canal [3,5]. Acute apical abscesses are a clear example of extraradicular disease dependent on intraradicular condition [3]. Once the bacterial load within the root canal is handled, the host defense could eradicate the extraradicular infection. Another example is the clinical healing of a sinus tract after endodontic procedures. When extraradicular infection is independent, intracanal disinfection procedures will be ineffective in eradicating the disease, leading to endodontic treatment failure [5].

Several clinical cases [6,7] demonstrated the presence of biofilms and areas of mineralization with a calculus-like appearance on the external apical root surface. The microorganisms involved in an independent extraradicular infection may be self-sustaining [8]. They can create strategies to survive in an inhospitable environment by evading host defense cells and molecules, protecting themselves from the complement system, and resisting phagocytosis [5,9]. Endodontic success will only be achievable in these cases through apical surgery [5].

Recent studies [10,11,12] have enhanced comprehension of the microbiology of extraradicular biofilms, mainly focusing on the resistance mechanisms that enable microorganisms to survive outside the root canal system. Next-generation sequencing (NGS) studies have identified *Actinomyces* spp., *Propionibacterium* spp. [10], and Enterococcus faecalis [11] as the predominant bacterial species in persistent extraradicular infections. These species can form biofilms, helping them escape host immune responses and resist antimicrobial treatments [12].

Another crucial aspect to consider is the role of the host immune response in regulating extraradicular biofilm formation. Studies suggest chronic inflammation within periapical tissues can contribute to biofilm persistence by providing nutrients and forming an extracellular matrix for bacterial adhesion [9,13]. Moreover, the inflammatory exudate in the periapical lesion is rich in calcium and phosphate ions, which can cause the mineralization of biofilms into calculus-like deposits [14].

The lack of large-scale clinical trials that comprehensively evaluate extraradicular infections and biofilms makes case reports the most valuable source of evidence for understanding their clinical characteristics, microbiology, and treatment challenges. Clinical trials typically require large, homogeneous sample sizes and standardized diagnostic criteria, which are difficult to establish for extraradicular infections due to their low prevalence and variability in presentation. Moreover, most clinical trials focus on treatment outcomes rather than microbiological or histological confirmation of biofilms, limiting their ability to provide direct evidence of extraradicular biofilm presence.

In contrast, case reports offer thorough clinical, histological, and microbiological records, enabling a more accurate description of extraradicular infections. Many of these reports utilize advanced imaging (CBCT) [15], histobacteriologic techniques [3], scanning electron microscopy (SEM) [16], and molecular techniques (PCR, NGS) [5] to confirm biofilm presence, which large-scale clinical studies do not typically include. Furthermore, mineralized extraradicular biofilms are often identified only during apical surgery or histopathological analysis of extracted teeth, making them unsuitable for prospective clinical trials.

This article aims to contribute to our understanding of the causes of endodontic treatment failure by examining the clinical findings published in cases involving extraradicular biofilm.

## 2. Materials and Methods

### 2.1. Case Report Review

#### Selection of Case Reports

A search of PubMed, Web of Science, Scopus, Embase, and Science Direct was carried out until June 2023, using the keywords “extraradicular infection and/or wet canal and/or wet canals and/or extraradicular mineralized biofilms and/or calculus-like deposit”.

Two reviewers independently screened titles and abstracts, followed by a full-text evaluation based on predefined inclusion and exclusion criteria. Any disagreements regarding study inclusion were resolved through discussion, and when necessary, a third reviewer was consulted to reach a consensus.

The inclusion criteria for this review were as follows: (1) case reports documenting extraradicular infections associated with endodontic treatment failure, confirmed through diagnostic methods such as histobacteriology, SEM, or PCR; (2) studies focusing on patients with symptomatic or asymptomatic apical periodontitis, where extraradicular biofilm or calculus-like deposits were present on the apical root surface; (3) reports involving both male and female patients aged 18 to 60 years; and (4) articles providing detailed descriptions of adjunctive endodontic procedures, such as calcium hydroxide treatment or passive ultrasonic irrigation (PUI), used to manage persistent infections. Exclusion criteria included (1) studies not specifically addressing extraradicular infections or post-treatment apical periodontitis, (2) reports lacking confirmation of extraradicular infections by histological or molecular techniques, and (3) cases where extraradicular biofilm or calculus-like deposits were not observed. This approach ensured the inclusion of clinically relevant cases highlighting the challenges of managing extraradicular infections in failed endodontic treatments. Subsequently, relevant titles and abstracts were selected for final reading.

## 3. Results

**Figure 1:**
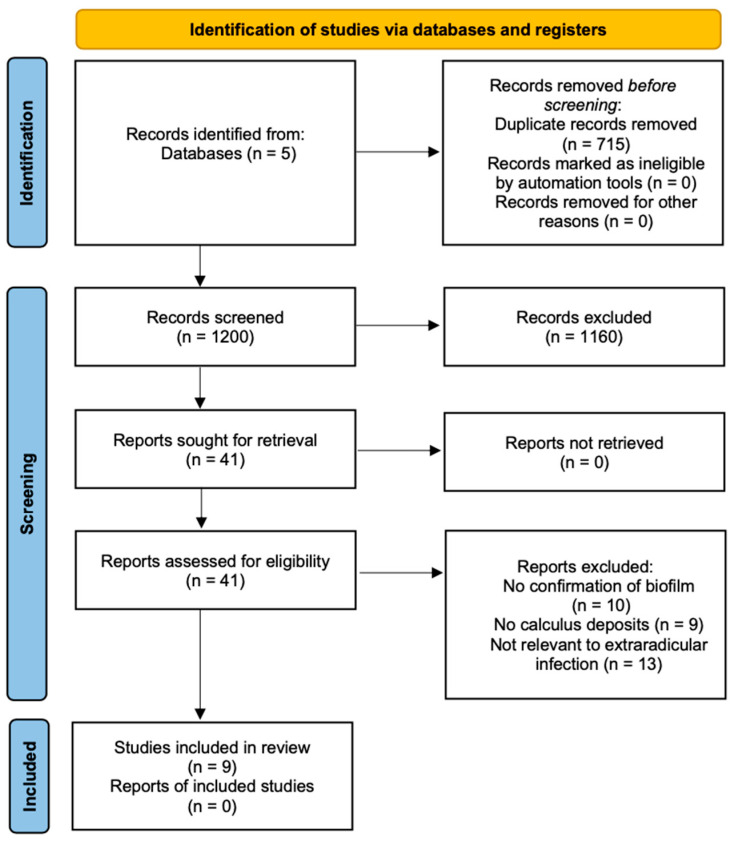
PRISMA flowchart of case report selection: 1200 records identified, 41 full-text reviews, 32 exclusions, and 9 case reports selected, totaling 15 clinical cases.

The search found 1200 studies in the five databases, and 41 relevant papers were carefully read. After applying the inclusion and exclusion criteria, nine case reports were selected. The reasons for excluding the other 32 articles are described in the flowchart (Figure 1).

Table 1 shows the nine chosen case reports [2,3,5,6,7,16,17,18,19] and the characteristics of cases treated as failed. Fifteen clinical cases with extraradicular infection were observed by complementary methods like histobacteriology, SEM, or PCR. Eight women and six men showed at least one case of extraradicular infection. These patients are between 18 and 60 years old, and a calculus-like deposit was observed in six patients (six teeth).

### 3.1. Characteristics of Included Cases and Analysis of Extraradicular Infections

#### 3.1.1. Demographics and Patient Characteristics

The patients analyzed in these studies range in age from 18 to 70. The gender distribution is relatively balanced, with male and female cases reported across different studies. However, there is no clear indication that gender significantly influences susceptibility to extraradicular infections.

Sinus tracts are commonly noted as a significant symptom. In nearly all cases, there is either a single sinus tract or accompanying pain and swelling. Notably, many instances lack evidence of periodontal probing, hinting at an endodontic cause, while others display additional periapical issues. Typically, the lack of periodontal probing suggests that these infections are likely extraradicular rather than a result of advancing periodontal disease.

#### 3.1.2. Root Canal Conditions and Periapical Diagnoses

Five patients (nine teeth) had asymptomatic apical periodontitis, and seven teeth presented with symptomatic apical periodontitis. Eight teeth showed post-treatment apical periodontitis, and nine patients showed a clinical sinus tract. No case displayed a periodontal pocket. Swelling was observed in five patients during the intraoral examination.

#### 3.1.3. Adjunctive Procedures and Treatment Approaches

Calcium hydroxide (CH) was the most frequently used intracanal medication, often applied for extended periods ranging from 1 week to 15 weeks. Its use for multiple weeks reflects the difficulty of eradicating extraradicular infections, as prolonged exposure may be necessary to achieve antimicrobial effects beyond the confines of the root canal. Notably, PUI and apical negative pressure (ANP) are adjunctive procedures used in some cases, particularly in more recent studies.

#### 3.1.4. Extraradicular Biofilm and Calculus Formation

A crucial aspect of the data was the identification of extraradicular biofilms and calculus-like deposits. In all reported cases, biofilm was observed in some form. Interestingly, there is variability in the presence of both biofilm and calculus, with some cases showing only biofilm, while others exhibit both. The cases also demonstrate that not all extraradicular infections exhibit calculus formation, reinforcing that while biofilms are a consistent feature, the extent of mineralization may depend on factors such as bacterial composition, duration of infection, and host response.

#### 3.1.5. Comparison Across Studies

A longitudinal comparison of the included case reports reveals an increasing emphasis on mechanically and chemically enhanced disinfection methods in more recent cases. Earlier studies (e.g., Tronstad et al. 1990) [19] primarily relied on CH and chemical agents such as quaternary ammonium compounds, whereas more recent cases incorporate PUI and ANP to improve disinfection outcomes.

Another noteworthy trend is the prolonged use of CH in cases of symptomatic extraradicular infection, with some cases requiring up to 15 weeks of intracanal medication. This suggests that in the presence of extraradicular biofilms, standard endodontic treatment alone may not be sufficient, and extended antimicrobial strategies are necessary.

## 4. Discussion

### 4.1. Etiology

The apical lesion is usually microorganism-free, but bacteria may invade the periapical tissues and establish an extraradicular infection [4,6]. Extraradicular infections have been considered one of the possible causes of post-treatment apical periodontitis [20]. However, bacteria have difficulty leaving the canal and establish an infection beyond the root canal’s limits. It is probable that, in some situations, this can happen and result in a persistent infection [5].

When it happens, the extraradicular infection usually extends the intraradicular infection [7]. It is difficult to determine when an extraradicular infection is dependent or independent of an intraradicular infection since most clinical case reports did not evaluate the bacteriological conditions of the apical canal (Table 2) [21,22,23,24].

Determining whether an extraradicular infection is dependent or independent of the intraradicular infection remains a significant challenge [3]. The only reliable method to establish this distinction is through apical surgery, provided that the apex of the tooth can be retrieved along with the associated lesion [3]. This allows for a meticulous histobacteriological analysis using serial sectioning techniques. By carefully examining these sections, it is possible to investigate whether there is a direct communication between bacteria inside the root canal system and those in the periapical lesion or adhered to the apex of the tooth [5]. However, even with this approach, identifying such a connection can be complex and may not always yield definitive conclusions [25].

In a histological study, Ricucci et al. [26] considered several endodontically treated teeth with apical periodontitis, and no cases of independent extraradicular infection were found. In a few cases where bacteria were observed outside the root canal system, there was a concomitant intraradicular infection.

However, some clinical case reports have recently indicated an independent extraradicular infection of the root canal [5,6], demonstrating that, although challenging, microorganisms can self-sustain in the inflamed apical tissues under certain circumstances, leading to endodontic treatment failure. It has been suggested that this situation typically occurs when microbes adhere to the apical root surface, forming biofilm-like structures [4,27].

Periapical actinomycosis has been proposed as a potential cause of extraradicular independent post-treatment apical periodontitis [28]. Studies on the prevalence of apical actinomycosis in cases of apical periodontitis report an incidence of approximately 2% to 4% [29,30,31]. Furthermore, other bacterial species in the periapical tissues [32,33,34] and their potential contribution to persistent apical periodontitis suggest that this pathological condition is not solely attributable to actinomycosis. Thus, insufficient evidence indicates that periapical actinomycosis can perpetuate inflammation and lead to post-treatment disease.

An emerging area of interest in extraradicular infections is the role of biofilm-associated virulence factors in sustaining periapical inflammation [24]. Pathogenic bacterial species within biofilms produce extracellular polymeric substances, which enhance microbial adhesion and protect bacteria from host immune responses [18]. Additionally, quorum sensing mechanisms regulate bacterial communication within biofilms, increasing resistance to antimicrobial agents and host defenses [25].

Studies indicate that certain bacteria, such as *Porphyromonas gingivalis* and *Fusobacterium nucleatum* [35], can modulate host immune responses by secreting proteases and toxins [36], resulting in prolonged periapical inflammation [37]. Furthermore, bacteria-derived lipopolysaccharides and exotoxins contribute to tissue destruction and bone resorption in periapical lesions [38].

Understanding biofilm-associated virulence factors may lead to developing targeted therapies that disrupt bacterial communication and biofilm integrity [39], opening new avenues for managing persistent extraradicular infections. Future studies should investigate quorum sensing inhibitors and biofilm matrix disruptors as adjunctive treatments to conventional endodontic therapy.

Although it is less likely to occur, biofilm may also be driven to periapical tissues along with necrotic dentin debris due to over-instrumentation [6] or sealer extrusion during root canal filling, establishing an independent extraradicular infection [5]. In such cases, bacteria can protect themselves from host defenses, allowing them to survive and sustain the inflammatory response.

**Figure 2:**
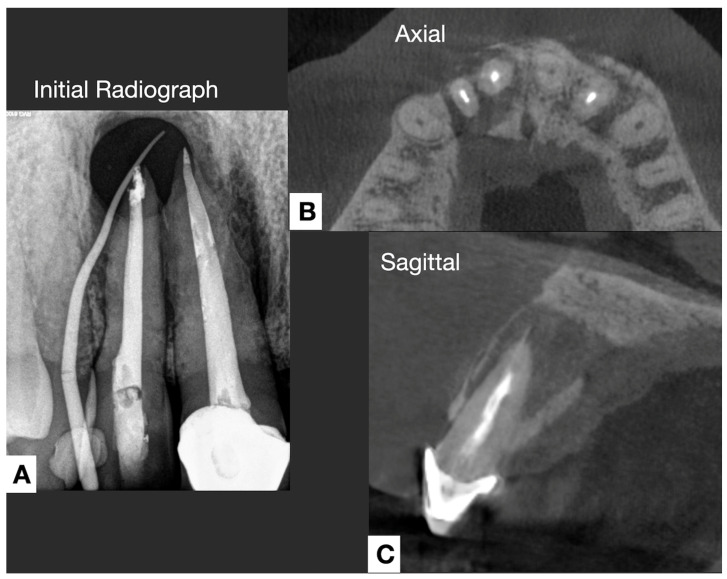
(**A**) Preoperative periapical radiograph of right maxillary central and lateral incisors (7 and 8) showing an extensive apical lesion encompassing the apex of both teeth and with the presence of sinus tract. (**B**,**C**) CBCT axial, and sagittal scans show a loss of bone and lingual cortical.

**Figure 3:**
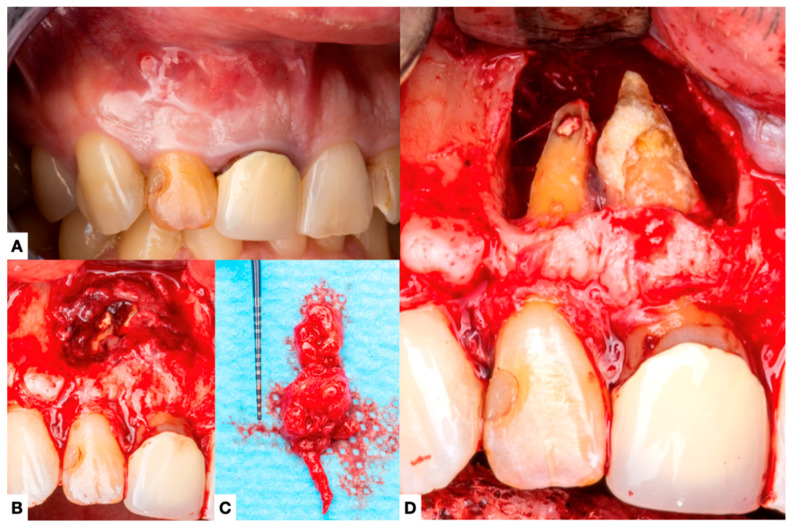
(**A**) Initial clinical situation with a sinus tract, a discolored tooth in the lateral incisor, and a misadjusted crown on tooth #8. (**B**) In the image after the elevation of the flap, it is possible to observe the presence of a large amount of granulation tissue, and in (**C**) after it has been wholly enucleated. (**D**) Clinical picture of the root apex showing a thick mass of calculus-like deposit on the central incisor.

**Figure 4:**
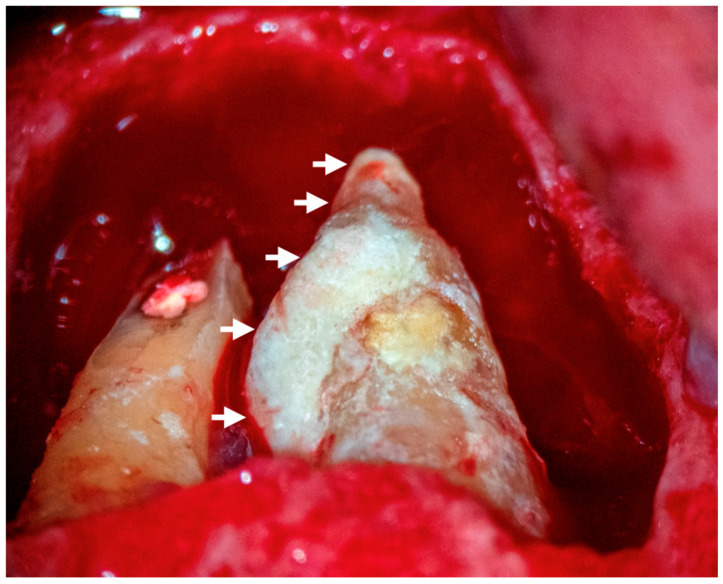
A magnification of the image in Figure 3D where a large mass of a deposit like-calculus, indicated by arrows, can be seen at the root surface of the maxillary central incisor. The tooth had no probing or periodontal pocket.

Other forms of extraradicular infection have been considered possible causes of persistent apical periodontitis. Some studies [4,20,26] indicate that the extraradicular biofilm may mineralize (Figure 2, Figure 3 and Figure 4). This statement is primarily based on the fact that none of the cases observed had periodontal pockets (Figure 2C and Figure 3A) reaching the apex of the tooth. Nevertheless, apical calculus and extraradicular infection were confirmed (Table 1).

Supra- or subgingival calculus is a bacterial biofilm mineralized by mineral ions provided by saliva or crevicular fluids, primarily composed of calcium phosphate mineral salts [40]. It cannot be determined whether the exact mechanism of calculus adherence occurs on the apical surface of the root; however, mineralization in these structures may imply that a similar process takes place [7]. Mineralization can occur in the extraradicular biofilm through several possible mechanisms. One potential source is inflammatory exudate and apical tissue fluids rich in minerals due to bone solubilization [7]. Additionally, long-standing sinus tracts might act as communication pathways between the apical area and the external environment, facilitating the movement of minerals and salts from oral fluids toward the apical lesion [17]. An extensive periodontal pocket communicating with the root apex could also contribute to the formation of apical calculus.

Fortunately, a study reported that the formation of extraradicular biofilms is rare, occurring in only 6% of cases, including both treated and untreated cases with apical periodontitis [4]. Another study found an extraradicular infection in 85% of patients with sinus tracts [20]. That same study noted that 59% of the extraradicular biofilms displayed calculus-like deposits [20]. Evidence suggests that the presence of a sinus tract before treatment is linked to a lower success rate [41], as it may indicate the presence of a well-established extraradicular biofilm resistant to standard endodontic therapy.

It is also essential to consider patient-related factors, such as systemic conditions (e.g., diabetes, immunosuppression) that may impair healing and increase the likelihood of persistent infection [42,43]. Personalized treatment planning, incorporating both microbiological analysis and patient health status [44,45], may improve clinical outcomes in challenging cases.

### 4.2. Clinical Signs and Symptoms

It is possible to observe that in several clinical case reports [3,17,18,19], the patients attend the office complaining of a sinus tract infection. Commonly, patients with a chronic apical abscess exhibit an extraradicular infection usually dependent on an intraradicular infection [20]. Generally, with the various intracanal disinfection strategies available, the sinus tract heals. From a clinical perspective, it is difficult to determine the cases in which we deal with an independent extraradicular infection. Nonetheless, it is crucial to note that when a case fails, a common finding is a sinus tract that does not resolve after several appointments [18,19] or, in some instances, may heal and re-emerge after treatment.

**Figure 5:**
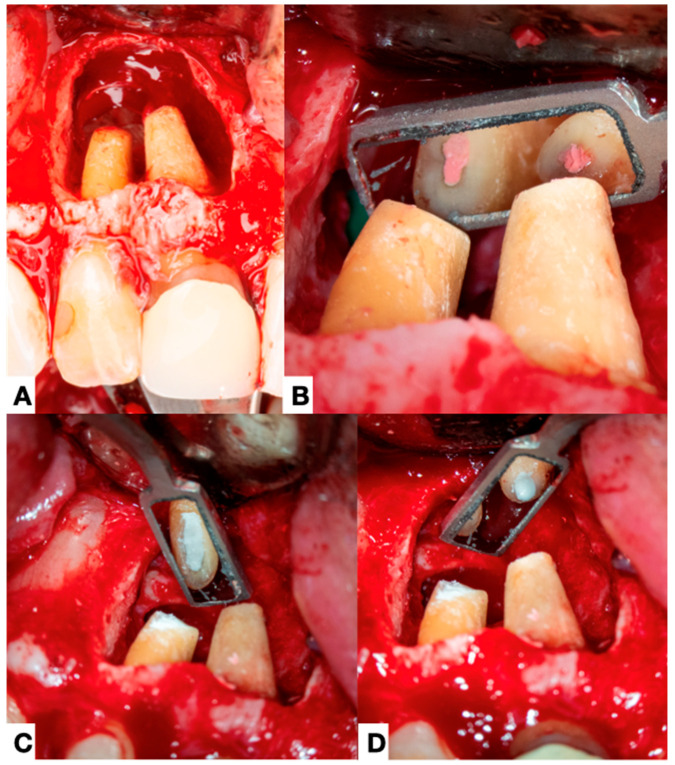
(**A**) Clinical images showing the situation of the two teeth (7-8) after granulation tissue removal and root surface cleaning. (**B**) View of the apex of both teeth after root apex resection and (**C**,**D**) retropreparation and retro root canal obturation.

**Figure 6:**
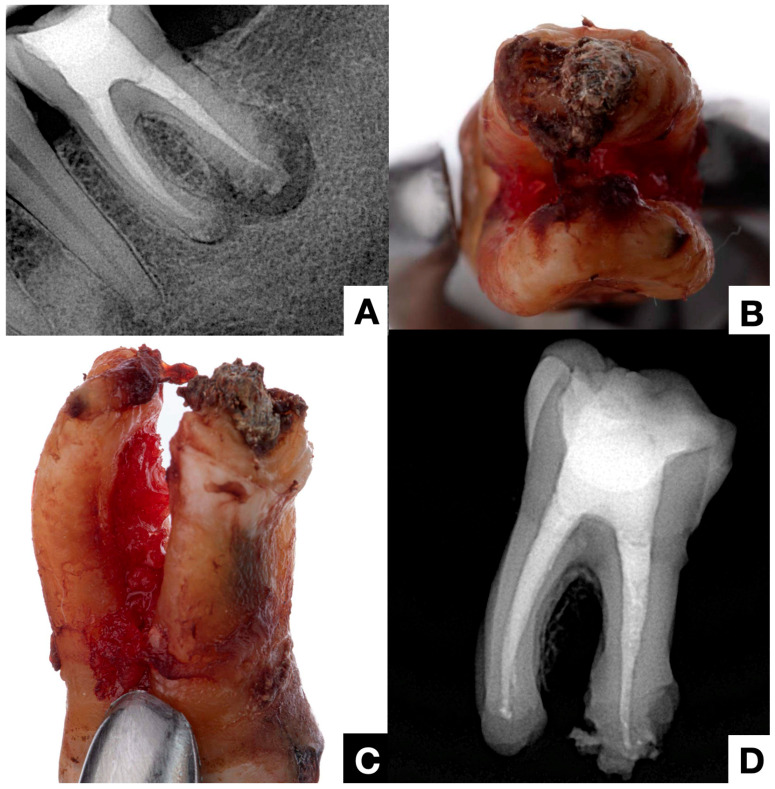
(**A**) Periapical radiograph of a previously treated tooth showing apical periodontitis, apical root resorption, and a radiopaque image on the distal root suggestive of apical calculus. Despite an adequate treatment, the patient experiences persistent pain, refuses further treatment, and opts for extraction. (**B**,**C**) Post-extraction images revealing extraradicular infection at the distal root apex. The tooth exhibited no periodontal probing. (**D**) Post-extraction radiograph showing more evident evidence of apical biofilm mineralization (clinical case courtesy of Javier Domínguez Bernal).

This review observed apical calculus in six cases (Table 1). When there is a long-standing extraradicular infection, this infection is likely independent of the intraradicular infection. Similarly, whether dependent or independent, the case will fail after any intracanal procedure and can only be resolved surgically (Figure 5), as its location will not respond to nonsurgical root canal treatment. Interestingly, a recent case report [7] clearly distinguished a radiopaque area on the root’s mesial or distal apical side, suggesting a calculus-like deposit. Therefore, apical calculus is likely observed in cases with extensive apical lesions and long-standing infections that do not respond to conventional endodontic treatment (Figure 6). However, this assertion is based on only one case, and future reports or clinical studies should confirm this observation.

On rare occasions, the extraradicular infection may present asymptomatically without a sinus tract [5]. The absence of clinical signs and symptoms could be related to low bacterial virulence. The host’s resistance may also have prevented symptoms and sinus tract formation [5].

Either way, it is crucial always to perform the endodontic treatment to control the intraradicular infection, even though extraradicular infection has been implicated in the failure of endodontic therapy, because the most unsuccessful outcomes can be attributed to persistent intraradicular infection remaining in the inaccessible apical areas [4,26,46], inside the root canal system.

Some patients complained about the presence of swelling, and drainage of purulent exudate through the root canal was observed when the treatment was performed [5,6,7], persisting despite several attempts to control the intracanal infection. The most common type of extraradicular infection is an acute apical abscess, characterized by purulent inflammation in the supporting tissues due to virulent bacteria migrating to the apical tissues [47]. The extraradicular infection is typically influenced by the intraradicular infection [26]; once the root canal is treated, the extraradicular infection is managed by host defenses and usually subsides. However, in certain cases, ongoing seepage of inflammatory exudate into the canal continues despite treatment, potentially leading to the appearance of a sinus tract likely due to the chronicity of the previous abscess [7], a condition known to result from the egression of pathogenic bacteria from the canal to the periapical tissue, causing persistent infection and subsequent failure of endodontic treatment.

### 4.3. Radiographic Assessment

Recently, the role of CBCT in detecting mineralized extraradicular biofilms has been hypothesized [21]. However, its ability to differentiate these biofilms from other mineralized structures remains limited [7], with evidence suggesting that histological or microbiological confirmation is necessary [23].

Radiographic evaluation is essential for decision-making in these complex cases. While periapical radiographs provide a two-dimensional view with limited diagnostic value, CBCT has emerged as a more effective imaging modality for assessing periapical infections [48]. In cases with extraradicular biofilm, radiographic patterns often include persistent periapical radiolucency, indicating endodontic treatment failure [4]. Additionally, cortical bone erosion or irregular resorption (Figure 6) has been observed in cases of chronic extraradicular infection [27]. Some cases exhibit radiopaque mineralized deposits, resembling apical calculus, which may indicate biofilm mineralization [7].

While CBCT provides high-resolution images of periapical bone destruction, its ability to differentiate biofilm structures from mineralized deposits remains limited [23]. Recent studies have explored fluorescence in situ hybridization (FISH) [49] and DNA sequencing [50] to confirm the presence of bacterial biofilms in medical samples.

Optical coherence tomography (OCT) is emerging as a promising real-time imaging modality for visualizing microbial biofilms [51]. OCT has proven to be a reliable predictor of biofilm presence and has been successfully applied in cases of chronic rhinosinusitis with nasal polyposis [52]. It enables noninvasive, high-resolution imaging of biofilms, offering a potential alternative to destructive histological analysis for observing extraradicular infections.

Future advancements in artificial intelligence (AI)-assisted imaging may further improve the accuracy of diagnosing extraradicular biofilms [53] by integrating machine learning algorithms with CBCT and OCT data [54].

### 4.4. Treatment of the Extraradicular Infection

Any attempt to control the infection will fail in all cases with independent extraradicular infection with or without apical calculus [6,7,17]. A common observation of the selected clinical cases is that, generally, any technique or procedure used to optimize disinfection is unsuccessful, and after several attempts, the canal is still wet, or the presence of an active sinus tract is still observed. In a minority of cases, it is possible to keep the signs and symptoms at bay [7], but after a time, the initial clinical situation reappears or worsens [3].

Extraradicular infections persist despite modern endodontic techniques due to biofilm resistance, limited irrigant reach, and host factors. Even with thorough endodontic disinfection, persistent inflammation and host immune modulation also contribute to treatment failure [55,56].

Even when strategies to optimize disinfection (PUI or ANP) [5,6,7] and intracanal medication for up to three months were used [7,18,19], the situation did not improve. This may relate to the inability of those techniques to eliminate the biofilm adhered to either the periradicular external root or located in the body of the lesion [4,20,26]. Additionally, bacteria may persist in anatomical complexities within the root canal system, inaccessible to disinfection procedures [57], or the intracanal medication may become inactivated by dentin, tissue fluids, and organic matter, all of which can limit its antimicrobial efficacy [58].

Antibiotics are ineffective in eradicating infections in persistent sinus tracts or wet canals [6,19]. In one case, the sinus tract persisted after using amoxicillin and metronidazole for 10 to 21 days [19]. In another case report, the patient was instructed to take amoxicillin/clavulanate for 14 days due to persistent exudation [6]. Subsequently, the canal remained wet, and apical surgery was scheduled. Thus, prolonged use of systemic antibiotics is likely unnecessary for treating this condition. Indeed, if an intraradicular infection perpetuates the disease, antibiotics will have limited efficacy because they may not reach the apical necrotic canal at sufficient concentrations. Moreover, if there is an extraradicular biofilm independent of the intraradicular infection, the antibiotic may not adequately eliminate this infection, leading to treatment failure, particularly in cases with apical calculus.

**Figure 7:**
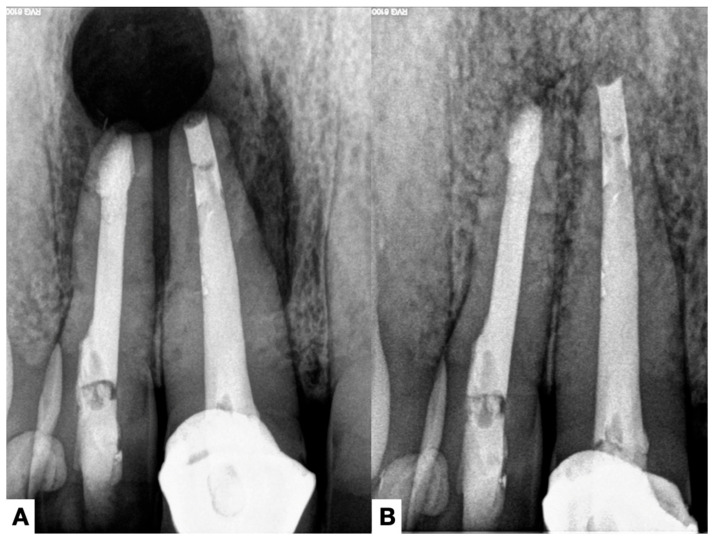
(**A**) Radiograph immediately after the microsurgery and (**B**) one-year recall periapical radiograph showing almost complete healing of the lesion.

From a clinical perspective, treatment should initially focus on controlling the intraradicular infection using all available tools. This includes supplementary strategies with the most significant possible volume and retention time of NaOCl [59], as well as the placement of intracanal medication [60] between appointments. If, after several sessions, there is any sign of persistent intra- or extraradicular infection, antibiotics should be avoided, and apical surgery should be considered (Figure 5), as it is the gold-standard treatment that can lead to success in these cases (Figure 7).

Long-term success rates of apical surgery in treating extraradicular infections vary based on infection type, the presence of biofilm, and case complexity. A systematic review and meta-analysis indicated that endodontic microsurgery has a high long-term success rate, averaging 77–91% over four years, which supports its effectiveness in managing persistent intraradicular or extraradicular infections [61].

Another viable treatment option for cases involving extraradicular biofilm is intentional replantation. This alternative allows for removing the apical portion contaminated by biofilm and adequate disinfection, retro-preparation, and retro-filling, facilitating bone remodeling [62].

Recently, new strategies have been developed to enhance the chances of controlling infections, primarily focusing on intraradicular infections. Since extraradicular infections often depend on existing intraradicular infections, improving intracanal disinfection techniques may also contribute to better management of extraradicular infections. Recent advancements in calcium hydroxide formulations have increased antibacterial efficacy and penetration into dentinal tubules, potentially enhancing disinfection [63]. Additionally, alternative irrigation systems, such as photon-induced photoacoustic streaming [64], have demonstrated promising results in removing biofilms from hard-to-reach areas, including the apical region.

Laser-assisted disinfection, particularly with Er: YAG and Nd: YAG lasers, has shown effectiveness in disrupting biofilms and eliminating bacteria [65,66], presenting a potential method for treating persistent intraradicular infections. Moreover, bioceramic materials have been suggested to seal root canal spaces and create an environment less conducive to bacterial survival [67]. However, despite these advancements, no current strategy effectively controls extraradicular infections associated with mineralized biofilms (apical calculus). In these instances, only microsurgical intervention remains viable for achieving bacterial eradication and periapical healing (Figure 7).

## 5. Conclusions and Future Directions

The complexity of extraradicular infections poses a significant challenge in endodontic treatment, especially when conventional therapies do not entirely eradicate bacteria. This review highlights the strong association between extraradicular biofilms and endodontic treatment failure, emphasizing the need for enhanced diagnostic and therapeutic strategies.

Future research should concentrate on the following areas:

- Development of novel antibiofilm agents specifically targeting extraradicular biofilms while preserving host tissue integrity.
- Advancements in imaging techniques, such as AI-assisted CBCT analysis, to enhance the detection of persistent periapical infections and mineralized apical biofilms.
- Longitudinal clinical studies evaluating the efficacy of non-surgical treatments, including photodynamic therapy, nanoparticle-based disinfection, and laser-assisted techniques for persistent apical periodontitis cases.
- Genetic and molecular studies aimed at identifying bacterial resistance mechanisms and potential biomarkers for predicting treatment outcomes.
- Exploration of bioceramic sealers and bioactive endodontic materials that promote periapical healing and inhibit bacterial adhesion, particularly in the apical region.

## Figures and Tables

**Table 1:** Demographic characteristics, signs and symptoms of the patients with extraradicular infection. AAP, asymptomatic apical periodontitis; SAP, symptomatic apical periodontitis; CH, calcium hydroxide; ANP, apical negative pressure; PUI, ultrasonic passive irrigation; female; M, male; W, weeks.

Author, Year	Teeth	Gender	Age	Signs/Symptoms	Root Canal Conditions	Periapical Diagnosis	Adjunctive Procedures	Extraradicular Biofilm and Calculus
Tronstad et al., 1990 [19]	25	F	60	Sinus tractNo periodontal probing	Treated	AAP	CH (3m), Formo, Amonio quater.	yes/no
Ricucci et al., 2005 [17]	13	M	22	Sinus tract, pain, no periodontal probing	Necrotic	SAP	CH (3w)	yes/yes
	5	F	51	Sinus tract, no periodontal probing	Treated	AAP	CH (4w)	yes/yes
Ricucci & Siqueira, 2008 [3]	7	F	32	Sinus tractPain	Necrotic	SAP	CH (5w)	yes/no
Su et al., 2010 [18]	24	F	33	Sinus tract and pain	Treated	SAP	CH (15w)	yes/no
	25	F	33	Sinus tract and pain	Treated	SAP	CH (15w)	yes/no
Signoretti et al., 2011 [16]	19	F	38	Sinus tractNo periodontal probing	Treated	AAP	None	yes/no
Ricucci, et al., 2015 [6]	24–25	M	35	Pain and swelling	Incompletely treated	SAP	CH (4w)	yes/no
	25	M	42	Swelling	Treated	AAP	CH (3w) and PUI	yes/no
	9	M	42	Pain and swelling	Necrotic	SAP	CH (8w) PUI	yes/yes
Ricucci, et al., 2016 [7]	7	M	29	Sinus tract, swelling and no periodontal probing	Necrotic	AAP	CH (12w)	yes/yes
	29	M	70	No pain	Treated	AAP	PUIANP	yes/yes
Ricucci et al., 2018 [20]	9	F	18	No pain	Necrotic	AAP	CH(2w) and PUI	yes/no
	10	F	18	Swelling	Necrotic	AAP		yes/no
Ricucci et al., 2023 [2]	10	M	61	Sinus tract	Treated	AAP	PUI, CH (1w)	yes/yes

**Table 2:** Clinical characteristics of dependent or independent extraradicular infections.

Characteristic	Dependent	Independent
Definition	Originates from and depends on an intraradicular infection; bacteria migrate from the canal into periapical tissues.	Persists independently of an intraradicular infection; bacteria are self-sustaining in periapical tissues.
Etiology	Typically occurs in cases of acute apical abscesses or chronic apical periodontitis where canal infection spreads beyond the apex.	Associated with extraradicular biofilm formation, where bacteria adhere to the root apex or surrounding periapical tissues without ongoing intraradicular infection.
Microbial features	Microorganisms are predominantly facultative and obligate anaerobes that originate from the infected root canal system.	Microbial composition includes Gram-positive bacteria (e.g., *Actinomyces* spp.), capable of surviving independently outside the root canal system.
Histologic findings	Bacteria are found within inflammatory infiltrates, often alongside immune cells attempting to eliminate the infection.	Biofilms form directly on the root surface, sometimes undergoing mineralization (apical calculus-like deposits), making them resistant to host clearance.
Response to endodontic treatment	Resolves with intracanal disinfection using effective irrigation.	Does not respond to intracanal disinfection alone, as bacteria survive outside the root canal system.
Clinical signs	Frequently presents with pain, sinus tracts, swelling, and purulent drainage due to the connection between intra- and extraradicular infection.	May be asymptomatic or persist despite successful root canal treatment, leading to treatment failure and chronic apical periodontitis.
Treatment approach	Requires root canal treatment and antimicrobial strategies to eliminate the intraradicular source of infection.	Often necessitates apical surgery to remove biofilms and mineralized calculus, as endodontic retreatment alone is ineffective.

## Data Availability

The data supporting this study’s findings are available from the corresponding author upon reasonable request.

## Abbreviations

The following abbreviations are used in this manuscript:

ANP	apical negative pressure
FISH	fluorescence in situ hybridization
OCT	optical coherence tomography
NGS	next-generation sequencing
PCR	polymerase chain reaction
PUI	passive ultrasonic irrigation
SEM	scanning electron microscopy
